# Real-world use of orphan medicinal products (OMPs) in rare disease (RD) patients: A population-based registry study

**DOI:** 10.3389/fphar.2022.940010

**Published:** 2022-09-30

**Authors:** Monica Mazzucato, Cinzia Minichiello, Andrea Vianello, Laura Visonà dalla Pozza, Ema Toto, Paola Facchin

**Affiliations:** ^1^ Veneto region Rare Diseases Coordinating Centre-Registry, Padua University Hospital, Padua, Italy; ^2^ Epidemiology and Community Medicine Unit, Padua University Hospital, Padua, Italy

**Keywords:** orphan drugs, rare diseases, population-based registry, real-world data, prescriptions

## Abstract

**Background:** Despite calls for the use of real-world data, the rare diseases (RD) treatment landscape suffers from a scarcity of data referred to orphan medicinal products (OMP) use at the population level.

**Objectives:** We aim to describe the characteristics and patterns of utilization of OMP in a sizable group of RD patients globally monitored by an area-based rare diseases registry located in the Veneto region, Italy, during a 3-year period (1 January 2019 to 31 December 2021).

**Methods:** A list of OMP (n = 60) was assembled for study purposes, according to extensive criteria with regard to the status of orphan designation and of national reimbursement decisions.

**Results:** OMP prescriptions involved 1,010 patients, corresponding to the 2.3% of all the patients monitored by the RD registry. Nearly one out of five (22.8%) was a pediatric patient at the time of the first prescription. OMP use interested a limited proportion (17.5%) of diseases approaching the rarity threshold, having a prevalence of less than five per 10,000, while individuals affected by these more common rare conditions represented 49% of all the patients receiving an OMP prescription. A clustering effect of OMP use was observed in selected groups of diseases, mainly, neurological, hematological, and hereditary metabolic ones. Medication plans including an OMP show in the 45.9% of the cases a high level of complexity, both in terms of nature and number of co-prescribed treatments. Off-label use interested 15.3% (n = 155) of all the RD patients with at least an OMP prescription during the study period.

**Conclusion:** Data collected in a real-world setting through population-based registries globally monitoring RD patients, including related medication plans, have the potential to identify which diseases, and thus patients, have less benefit from the advent of OMP so far. Furthermore, in the rapidly evolving RD therapeutic landscape, they can help understand which therapeutic areas are most in need of investment to address existing unmet care needs.

## Background

The US and the European Union (EU) have implemented legislations to stimulate the development of drugs for patients suffering from rare diseases (RD) (European Commission 1999; European Commission 2000, National Institute of Health, 2022). Thanks to these incentives, an increasing number of medicinal products have received the orphan designation and market approval. It has been demonstrated that the orphan status is a strong predictor of reimbursement, having orphan medicinal products (OMP) a more easy access to the market than innovative drugs for more common diseases (Dupont and Van Wilder, 2011). According to a recent study, both the disease indication and the type of EMA approval have an impact on reimbursement decisions, showing country specificities. A outweigh of positive decisions for products having oncological indications has been observed in some countries. In other ones, including Italy, conditional approval significantly decreased the chance of reimbursement (Malinowski et al., 2018). The high costs of orphan drugs and innovative cell and gene therapies progressively available on the market and their increasing financial impact on healthcare systems have stimulated a debate on pricing and reimbursement mechanisms and generated proposals for the development of new assessment systems (Hughes-Wilson et al., 2012; Jönsson et al., 2019; Zimmermann et al., 2021). Advanced therapy medicinal products reimbursement status differs across the EU countries, with the exception of CAR-Ts (Ronco et al., 2021). A more tailored assessment of economic aspects of these therapies has been advocated, considering the heterogeneity of these products and of the target population (Simoens et al., 2022). In 2020, the European Commission carried out an evaluation of the legislation governing medicines for rare diseases and pediatric patients. This initiative identified strengths and weaknesses of the OMP Regulation 141/2000 (European Commission 2020a) and has been followed by the launch of an open consultation in order to widely collect contributions on suggested amendments to the legislation in force (European Commission 2021). Since the enactment of the OMP Regulation, annual designation applications have almost tripled and annual OMP authorizations have increased from three in 2001 to 19 in 2021 (European Medicines Agency, 2021). Nevertheless, consistent differences in accessibility and availability of these products for patients across member states still exist. Sarnola et al., 2018 assessed the availability of orphan medicines in outpatient care in 24 European countries. According to this study, most of the analyzed countries did not implement special regulations or policies for assessing the reimbursement status or pricing of orphan medicines. Moreover, the number of available orphan products varied significantly across countries (Sarnola et al., 2018). In particular, an equity gap between Eastern and Western Europe in terms of the number of accessible orphan products and of related spending exists (Zelei et al., 2016; Szegedi et al., 2018; Malinowski et al., 2019). Even when investigating RD patients’ access to biotechnological drugs, a similar gap has been reported (Kamusheva et al., 2018). Inequalities are even more evident when considering a global perspective (Chan et al., 2020). In addition, it has been estimated that only a small fraction of the known rare diseases (5%) have an authorized treatment available, and even when available, these therapeutic options are not always able to significantly modify the natural disease history (European Commission 2020b; Tambuyzer et al., 2020). So far, most of the available literature on orphan drugs has focused on regulatory and economic issues (Abdallah et al., 2021; Tsigkos et al., 2021). After almost 4 decades since the approval of the orphan regulation in the US and after 2 decades from the approval of the European one, data on the real-world use of OMP are strongly needed to shed some light on this complex and rapidly evolving scenario and to possibly shape future directions. Real-world data (RWD) included in electronic health records, claims data, and prescription databases can strongly support regulatory decision making for rare diseases (Breckenridge et al., 2019). In addition, registries represent valuable data sources. Although some operational, technical, and methodological challenges exist (McGettigan et al., 2019), potential solutions to address them so that collected data can adequately support regulatory assessments have been identified (Cave et al., 2019). Wu et al. provided three concrete examples to illustrate how RWD can be used to generate real-world evidence to support regulatory decisions on treatments for rare diseases (Wu et al., 2020). Kölker et al. focused on inherited metabolic diseases, demonstrating how patient registries can fulfill multiple purposes. The study provided examples of the use of data from already established registries to carry out, in collaboration with EMA, post-authorization safety studies on two orphan drugs: Cystadane^®^ (betaine anhydrous) and Ravicti^®^ (glycerol phenylbutyrate) (Kölker et al., 2022).

In Italy, most of the EMA authorized orphan drugs are fully reimbursed by the National Health System (NHS) according to well-defined criteria. Moreover, patients can access OMP in the absence of a marketing authorization through other possible pathways established per law (i.e., regulating compassionate use). A dedicated special fund can be used as well to access OMP free of charge. In this case, access occurs under specific conditions and following an authorization issued by the Italian Medicines Agency (AIFA) on an individual basis (Italian Medicines Agency 2022). In Italy, OMP are subject to the same health technology assessment (HTA) and pricing procedure applied to non-orphan drugs (Young et al., 2017; Jommi et al., 2021). To accelerate their availability at the local level, the pharmaceutical company holding an OMP marketing authorization can apply to the Italian Medicines Agency (AIFA) for pricing and reimbursement as soon as a positive opinion of the Committee for Medicinal Products for Human Use (CHMP) is obtained. Once the pricing and reimbursement process is concluded, access to OMPs finally depends on regions, which are accountable for the healthcare budget. Prescription can be limited at the regional level to selected specialist centers, especially for therapies with rare indications, for which expert centers have been already officially designated. Additional managed market entry agreements can be put in place following budget impact analyses carried out by regional health authorities. We sought to provide a snapshot of the real-world use of OMPs using a population-based registry collecting data on all the treatments prescribed to a consistent group of RD patients, followed by officially labeled expert centers. This retrospective study is based on the analysis of OMPs prescribed in the monitored population according to their orphan designation status, therapeutic indication, reimbursement status, on-label vs. off-label use; the analysis of RD patients receiving an OMP prescription according to their age class and diagnosis (defined using the Orphanet nomenclature of rare diseases); and the analysis of the medication plans including at least an OMP prescription, according to their composition (number and nature of prescribed co-treatments).

## Methods

The Italian healthcare system is a universal, region-based public system. The first legislation on rare diseases was issued in 2001; recently, a RD law further supporting patients’ care, research on these conditions, and OMPs production has entered in force (Ministero della Salute 2001; Gazzetta Ufficiale 2021). The Italian RD organization is based on the following pillars: a national list of diseases, whose affected patients are entitled to specific benefits; the identification of regional/interregional centers of expertise for RD, responsible for patient diagnosis, treatment, and follow-up; and a RD epidemiological surveillance system, based on population-based regional/interregional registries. A common minimum data set is provided by all RD regional registries to the national one, established at the Italian National Institute of Heath (ISS) (Istituto Superiore di Sanità 2022). We based the present work on data derived from the RD registry ongoing in the Veneto region (4.9 million inhabitants), northeast of Italy, since 2002. The registry (VR-RDR), already described elsewhere (Mazzucato et al., 2014), is based on a multi-source web-based information system combining aspects of a population-based registry with aspects of a more clinically oriented registry. It connects the RD centers of expertise *via* a protected network to all the hospital pharmaceutical services and the local health units of the Veneto region and of other seven Italian regions. Patients’ enrollment in the registry occurs when clinicians working in the labeled RD centers perform a diagnosis of one of the rare diseases included in the national list. Of note, currently seven healthcare providers located in the Veneto region are full members of the European Reference Networks (minimum 1 to 23 ERNs) (European Commission 2022). The national list of RD was expanded in 2017, including a consistent number of additional rare disease entities (Gazzetta Ufficiale, 2017). The level of diagnosis detail adopted in the VR-RDR is higher than that of the national list, which is established for reimbursement purposes. In the VR-RDR each rare disease is described using the following codes: ICD9-CM, ICD-10, MIM, and ORPHAcodes. For the present study, we considered RD entities monitored by the VR-RDR and described by ORPHAcodes, according to the Orphanet nomenclature file issued on July 2021 (Orphanet 2021). Furthermore, for analyses per classification group, we considered the contents of the linearization file, in which a preferential medical specialty is attributed to every clinical RD entity. For the analysis of the disease distribution per prevalence class, we used the Orphanet data and applied the methodology described in Nguengang Wakap et al., 2020. Over the years, specific forms have been developed in the registry to manage the prescription, procurement, and delivery of treatments to RD patients. Among them, pharmaceutical products, galenicals, medical foods, medical devices, and all the marketed pharmaceutical products are made available in the registry, together with their Anatomical Therapeutic Chemical (ATC) description (World Health Organization 2022). Furthermore, products available abroad can be prescribed as well based on a regularly updated list. Treatments prescribed according to appropriateness criteria through the RD registry and essential for patients’ care are delivered free of charge. Specific modules supporting the OMP prescription have been developed, allowing the collection of clinical data and long-term monitoring, even when treatment delivery takes place in the home-care setting. The Italian Medicines Agency (AIFA) periodically provides a list of orphan medicinal products. The criteria for including a product in the list have been modified in 2019 because of the definition of new pay-back mechanisms. We based our analysis on the last available OMP list issued by the Italian Medicines Agency (AIFA) in December 2021 (Italian Medicines Agency 2022). Currently, this list includes medicinal products having an active orphan designation and assigned to class H or A of reimbursement (class H includes medicines reimbursed only in hospital settings and class A includes drugs reimbursed also in the retail market). As drug inclusion in the list is partially based on reimbursement criteria, we additionally considered in our analysis the medicinal products with an orphan designation included in the EU Community Register, independently from their reimbursement status and from the termination of their period of market exclusivity (hereafter, past-orphan drugs). We have excluded in the present analysis authorized OMP that have been withdrawn by the manufacturer from the EU Community Registry. We have considered OMP for which at least one prescription was available during the study period in the monitored RD patients’ population. According to these criteria, a list of medicinal products (n = 60) was assembled (available as Supplementary Material). The study population includes residents of the Veneto region diagnosed with one of the RDs included in the Italian list and having received at least one prescription of an OMP, as defined before, performed by clinicians working in RD expert centers during the period from 1 January 2019 to 31 December 2021. For the analysis of the pharmaceutical products additionally prescribed to OMP, we referred to the Anatomical Therapeutic Chemical (ATC) classification system. Statistical analyses were performed using the SAS package, rel. 9.4 (SAS Institute Inc., Cary, NC, USA). Tables and figures were produced using Microsoft Excel, Office 2013 (Microsoft, Redmon, WA, USA).

## Results

### OMP list

**FIGURE 1:**
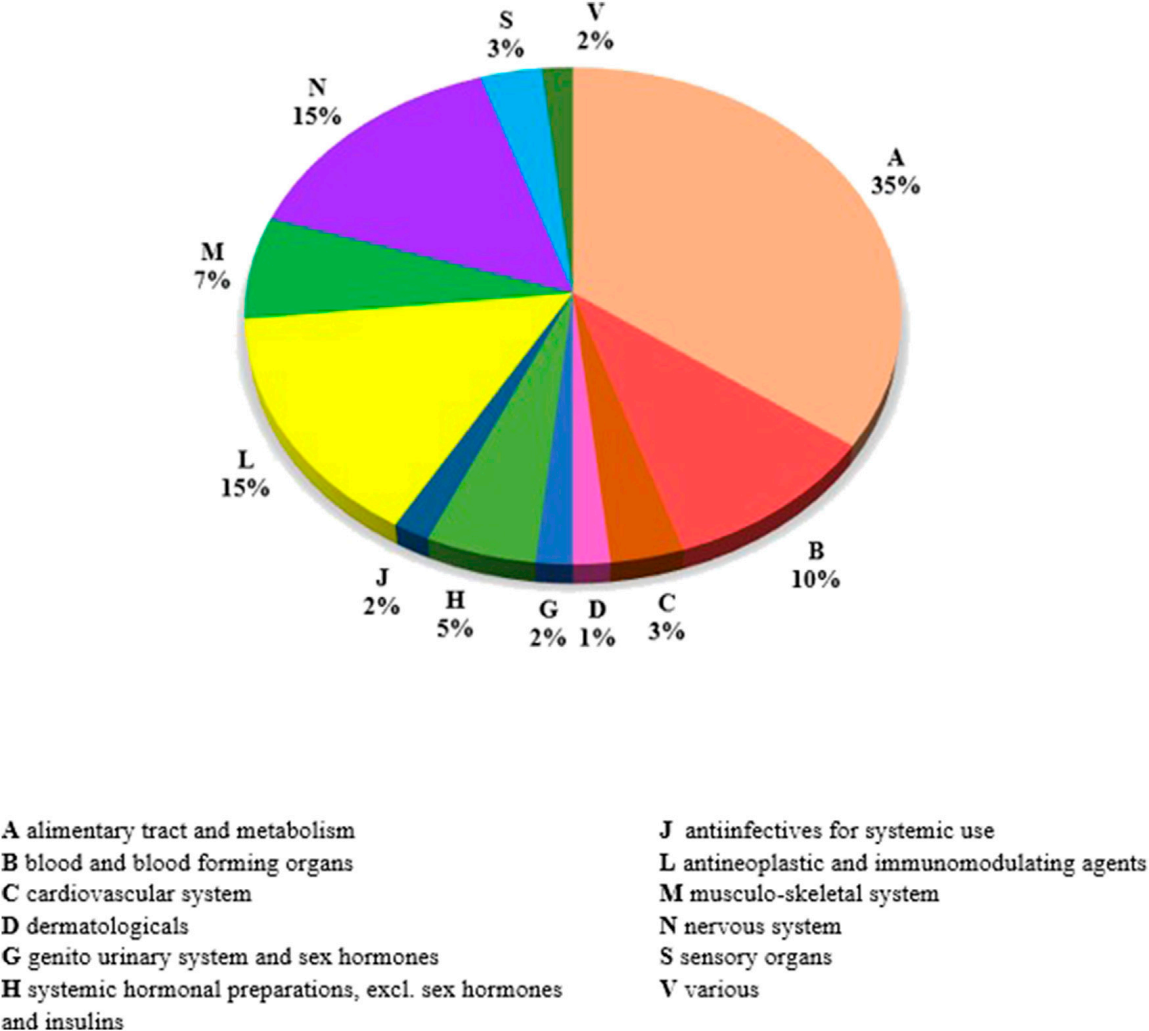
Distribution of medicinal products of the OMP list per first level of ATC classification.

The list of orphan medicinal products assembled for the present study purposes includes 60 different products. Of these, at 31 December 2021, 25 had a terminated market exclusivity period, defined as “past-orphans”, while 35 (58%) had an active orphan designation. The distribution of these medicinal products per ATC first level class shows that nearly one out of three belongs to the A-alimentary tract and metabolism class (35%). The others are described by the N-nervous system class (15%), by the L-antineoplastic and immunomodulation class (15%), and by the B-blood and blood-forming organs class (10%) (Figure 1). At 31 December 2021, the great majority of the medicinal products considered (n = 54) fall in classes H or A of reimbursement (fully reimbursed), while six were not directly reimbursed by the National Health System. The list of OMP considered in the study is composite, as it includes repurposed active substances such as Raxone^®^ (idebenone), Siklos^®^ (hydroxycarbamide), and Chenodeoxycholic Acid Leadiant^®^ (chenodeoxycholic acid), as well as highly innovative biotechnological products such as Onpattro^®^ (patisiran sodium), Crysvita^®^ (burosumab), Spinraza^®^ (nurinersen), and Zolgensma^®^ (onasemnogene abeparvovec). Nearly half (n = 27, 45%) of the OMP considered in the present study are biological/biotechnological products.

### Patients and prescriptions

**FIGURE 2:**
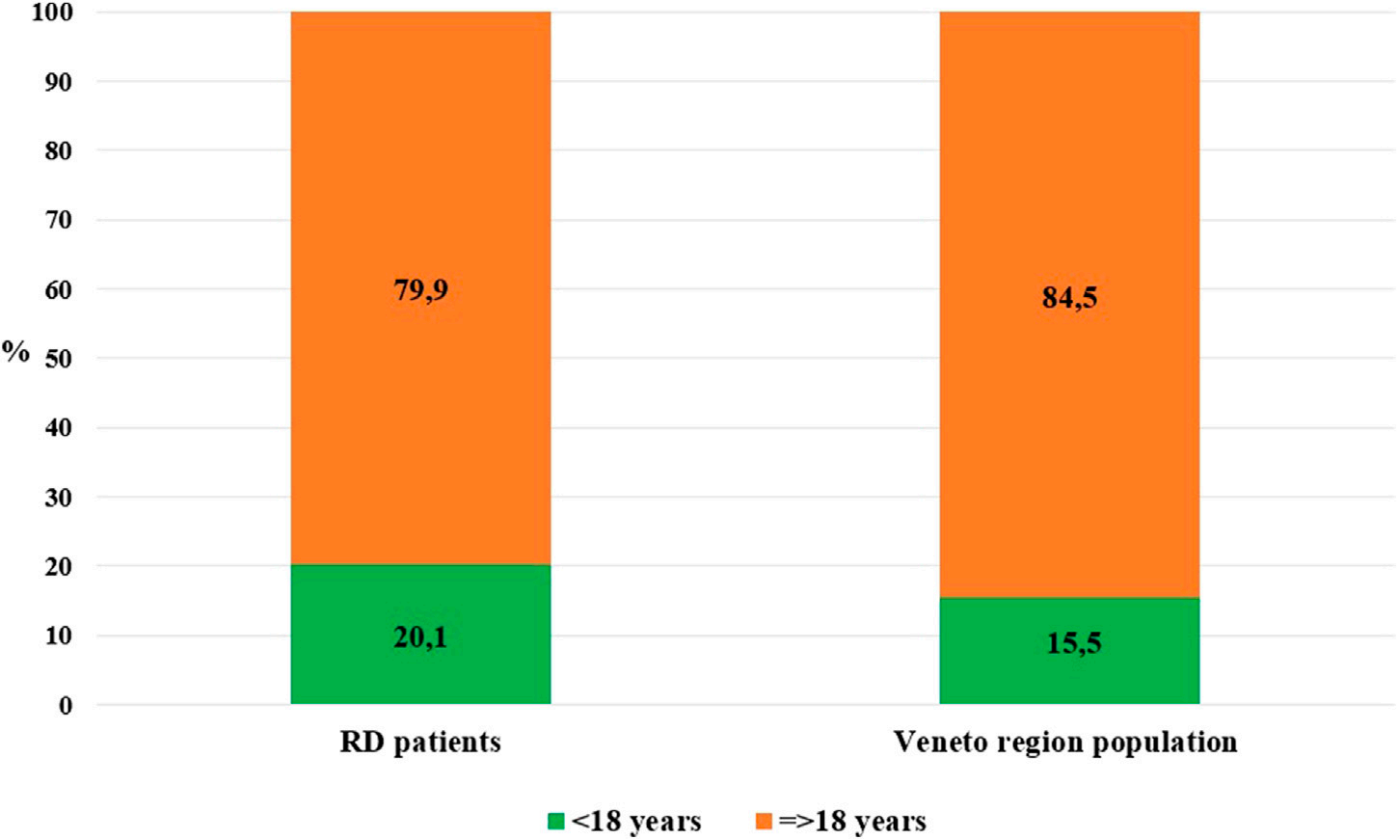
Distribution per age class: Veneto region population vs. RD monitored population (year 2021).

**FIGURE 3:**
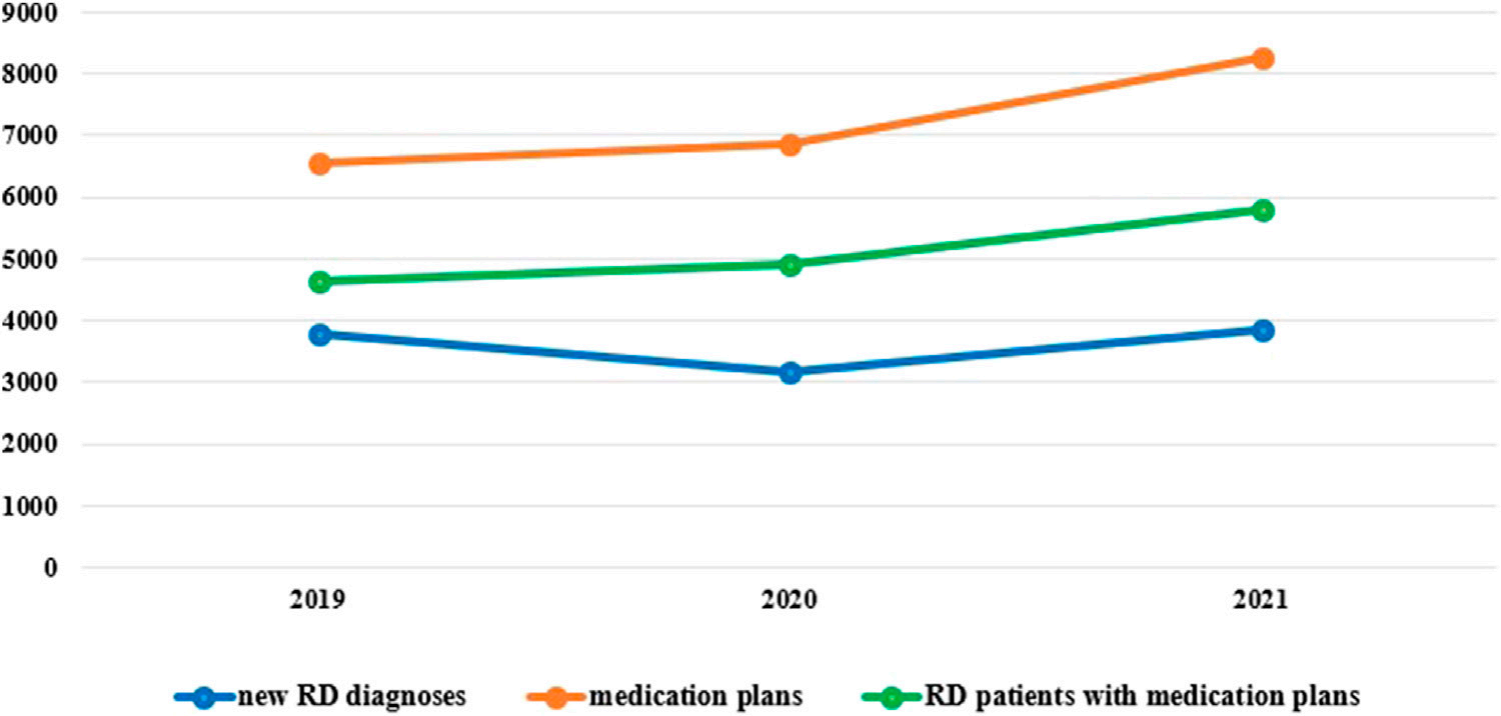
New RD diagnoses, RD patients with medication plans and medication plans, Veneto region, years 2019-2021.

**FIGURE 4:**
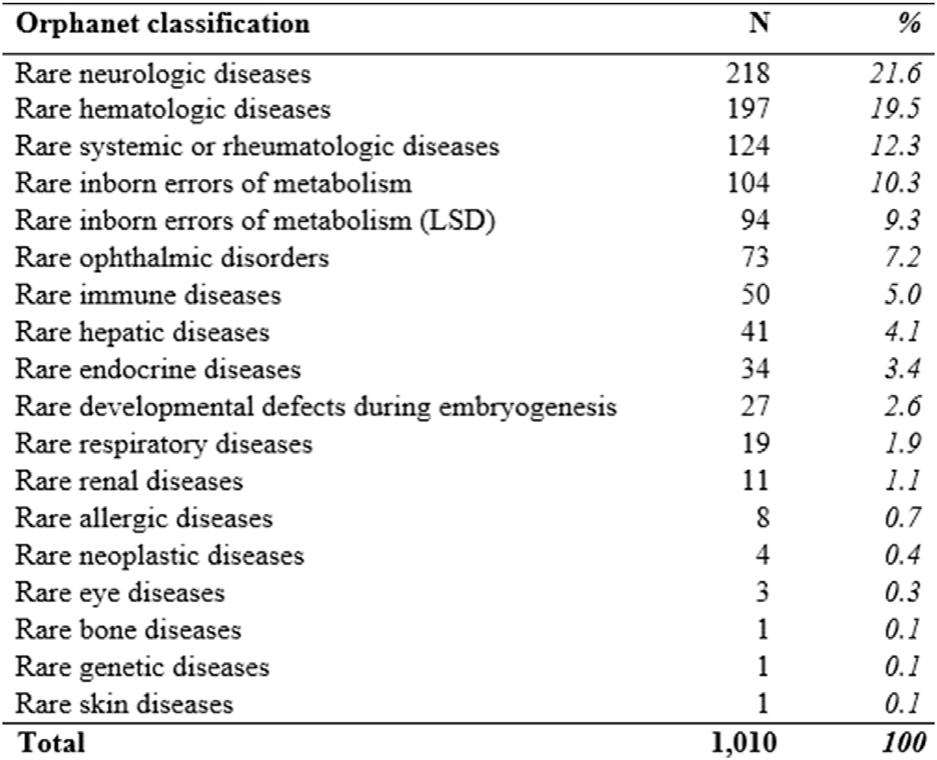
Diagnoses’ distribution of RD patients receiving an OMP prescription according to the Orphanet classification (preferential parent). LSD (Lysosomal storage disorders).

**FIGURE 5:**
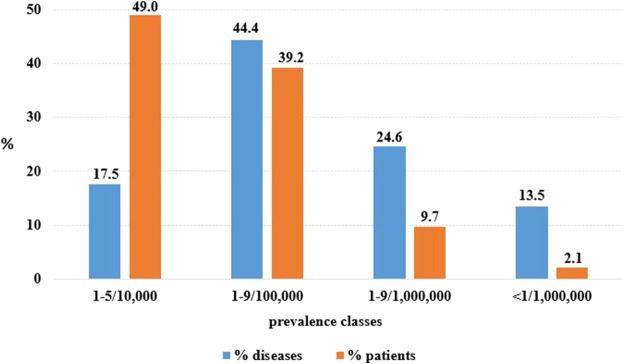
OMP use per diseases entities and rare disease patients according to disease point prevalence classes (prevalence data source: Orphanet DB).

**FIGURE 6:**
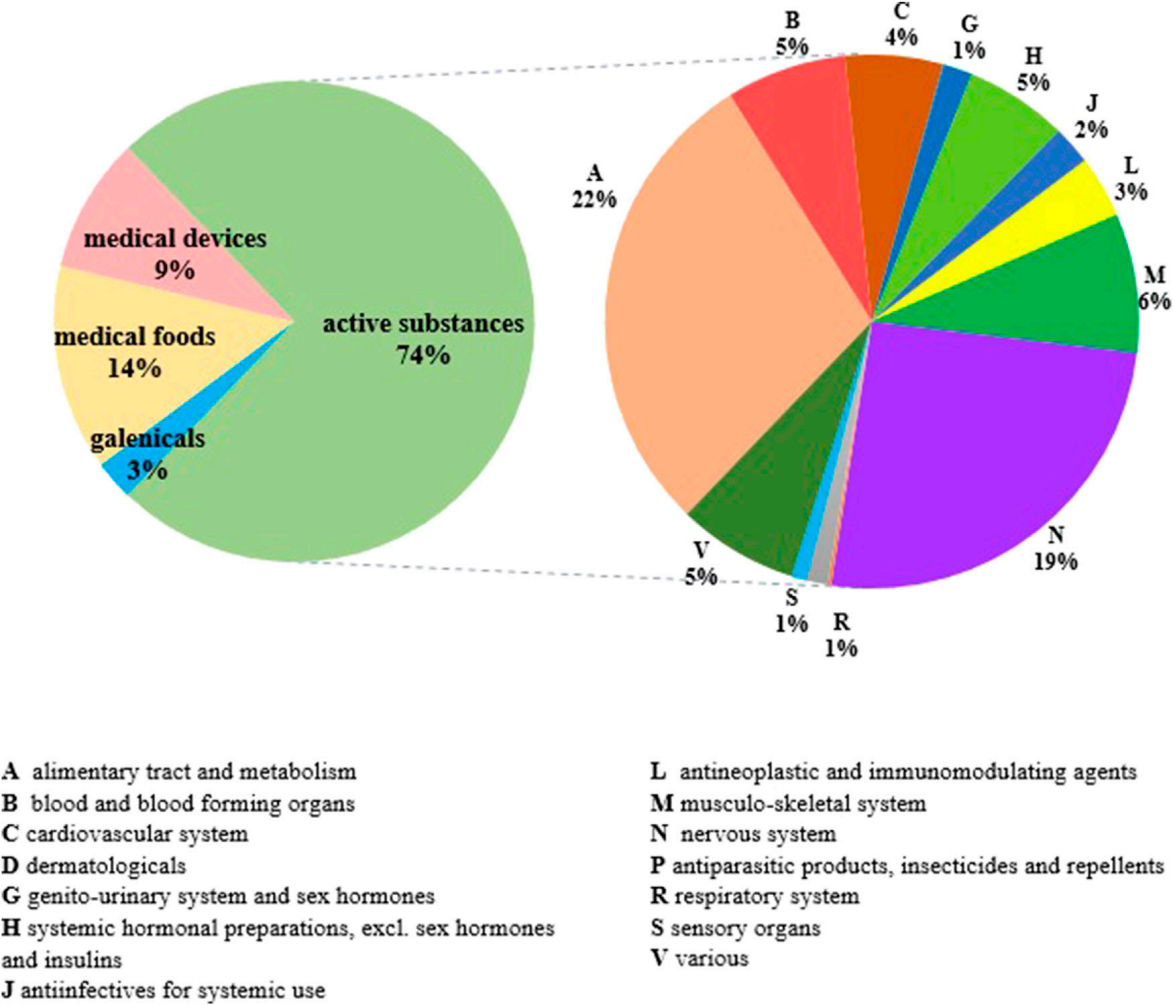
Distribution of medication plans including five or more cotreatments per treatment typology and per active substances ATC class (*n* = 544).

**FIGURE 7:**
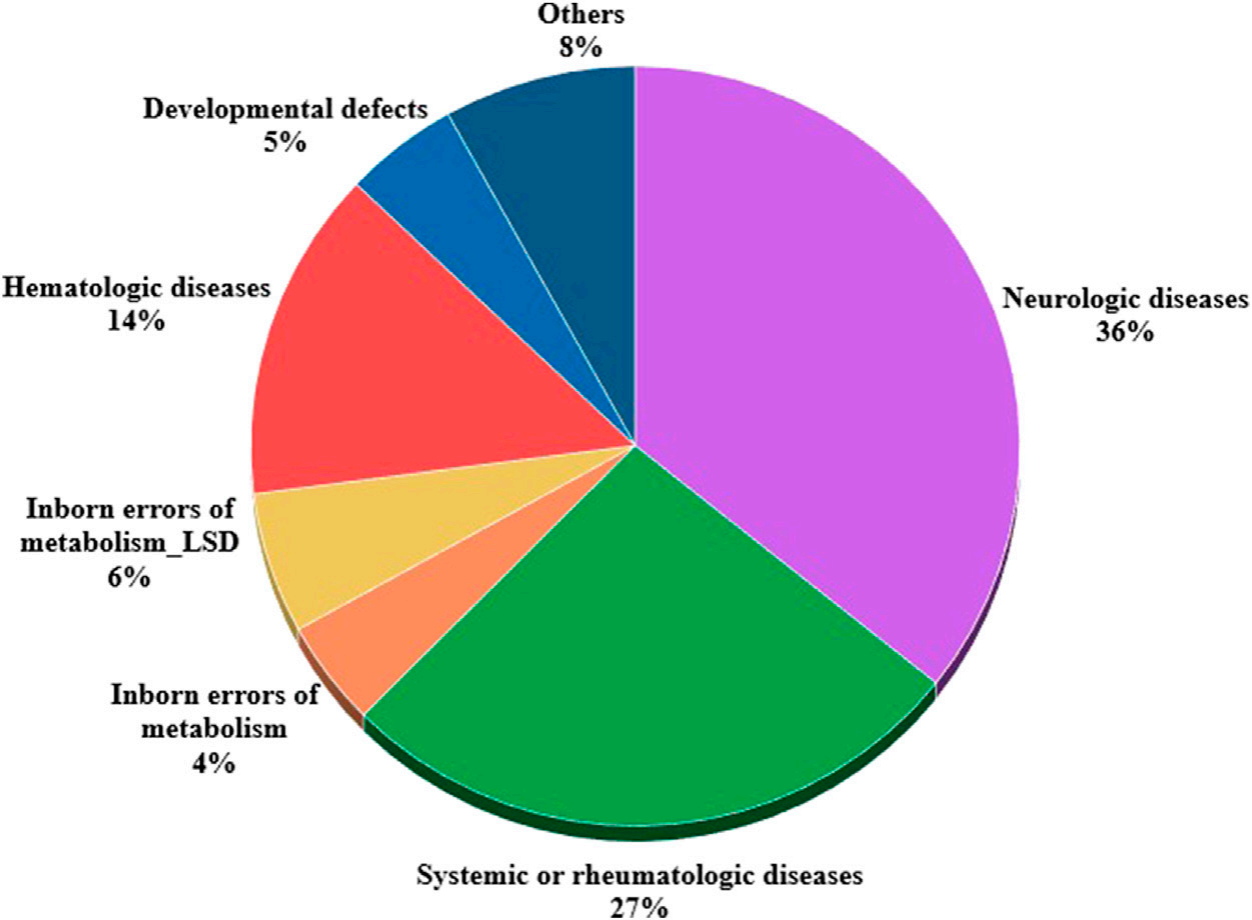
Distribution of OMPs used off-label per patients' disease according to Orphanet classifications (*n* = 333).

**FIGURE 8:**
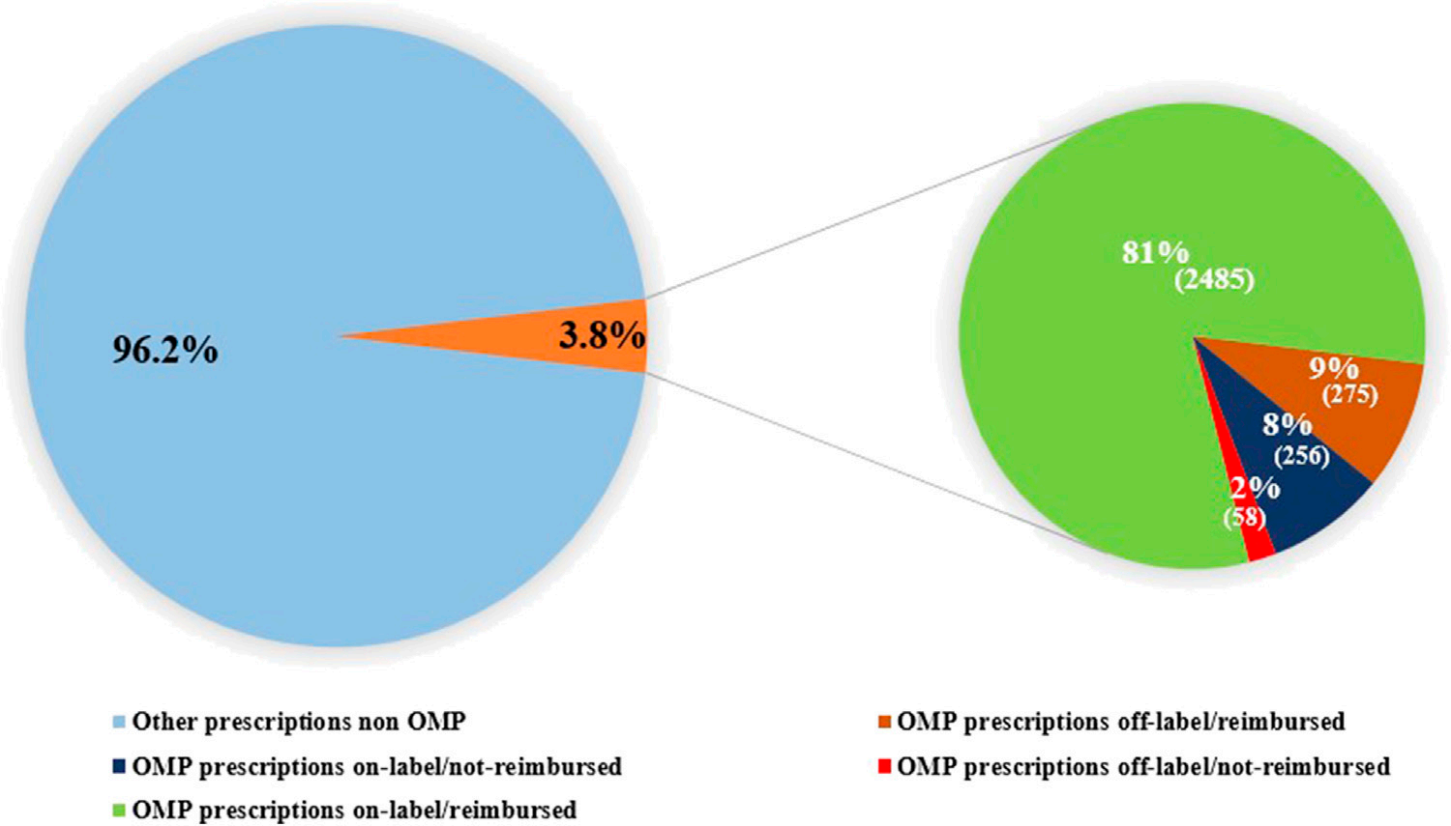
Distribution of OMP prescriptions per OMP use (off-label vs onlabel) and reimbursement status.

At 31 December 2021, 4,869,830 inhabitants were resident in the study area and 15.5% of them were under 18 years of age (Italian National Institute of Statistics, 2021). Among the monitored population, there were 42,910 individuals diagnosed with one of the rare diseases included in the Italian list. Of these, 20.1% were pediatric patients (Figure 2). During the study period (01.01.2019–31.12.2021) 80,091 prescriptions were included in 27,909 medication plans compiled by clinicians working in RD expert centers for 9,146 patients. The number of new RD diagnoses, medication plans, and RD patients with a medication plan registered during the study period is presented in Figure 3. As expected, in 2020, a decrease in the number of RD incident reported cases occurred, due to the pandemic impact on the healthcare system. However, in 2021, an overall increase in the activity of RD centers in terms of both their diagnostic and care capacity can be observed. Considering all the registered medication plans, there have been 3,074 prescriptions of OMP during the study period, corresponding to the 3.8% of the total, either for approved or off-label use. Overall, OMP prescription involved 1,010 patients, corresponding to the 2.3% of all the patients monitored and to the 11% of the patients for whom a medication plan was available in the registry during the study period. Among these patients, 602 (59.6%) had a prescription of a product having received an orphan designation but whose period of market exclusivity has expired (past-orphans). Considering all the prescriptions including an OMP (either with an active designation or past-orphan), 10.2% included products which were not directly reimbursed by the NHS, prescribed either for approved indications or for off-label use. Of the 1,010 patients having received at least an OMP prescription during the study period, 22.8% were in their pediatric age at the time of the first prescription. The diagnoses distribution of all patients receiving an OMP prescription is presented in Figure 4. It derives from the diagnoses recorded in the registry for each patient, using ORPHAcodes, and considering the corresponding preferential parent according to the Orphanet classification system. The vast majority of patients fall into the following three groups of the classification: rare neurological diseases (21.6%); inborn errors of metabolism (19.6%), mainly, lysosomal storage disorders; and rare hematologic diseases (19.5%). We analyzed the distribution per prevalence class of the diseases diagnosed in patients having received an OMP prescription (Figure 5). Prevalence data were extracted from Orphanet (Orphanet 2021). OMP prescription occurred in 17.5% of the cases for diseases falling in the most common prevalence range (1–5 per 10,000) and diagnosed in nearly half (49%) of the patients treated with OMP in our population. In 38% of the cases, OMP prescription occurred for very rare diseases (prevalence of less than 9 per million inhabitants), diagnosed in a minority (11.8%) of all OMP users. We sought then to investigate medication plans composition in terms of complexity. Considering medication plans with an OMP prescription, nearly half (n = 1,439, 54.1%) included additional prescribed treatments. Of note, nearly one out of five medication plans (n = 544, 20.4%) included five or more co-treatments, corresponding to a global amount of 4,394 prescriptions registered during the study period. The distribution per treatment typology of these highly complex plans is presented in Figure 6. The great proportion of OMP co-treatments are active substances (74%). Their distribution per ATC class shows that nearly half fall into the two following ATC classes: A-alimentary tract and metabolism class (22%) and N-nervous system class (19%). The other prescribed active substances are almost equally distributed across all the remaining ATC classes. Other co-prescribed treatments are medical foods (14%), medical devices (9%), and galenicals (3%). In total, 29 (48.3%) out of 60 OMP considered in the present study have been prescribed off-label, corresponding to nearly one out of 10 prescriptions both in the pediatric and adult population (12.1% and 10.3%, respectively). Patients receiving OMP off-label prescriptions were 155, corresponding to 15.3% of all patients with at least an OMP prescription during the study period. Most of the off-label prescriptions were for patients affected by rare neurological diseases (36%) and by systemic or rheumatologic diseases (27%), as shown in Figure 7. The distribution of OMP per off-label use and reimbursement status is presented in Figure 8. A great majority of OMP prescriptions were formulated according to the approved indication and have been fully reimbursed. A minority were on-label non-reimbursed uses (8%), namely, orphan products assigned to a non-reimbursed class by the national regulatory agency (e.g., Scenesse® (afamelanotide) and Siklos® (hydroxycarbamide)).

## Discussion

The present study provides a snapshot of the real-world use of OMP supported by data collected by a population-based registry monitoring a consistent group of RD patients, highlighting the potential of this data source in identifying eligible and treated patients. According to our findings, OMP prescription involves a limited proportion (2.3%) of rare disease patients. This subgroup is even more limited (1%), if we consider OMP with an active orphan designation at 31 December 2021. This percentage increases up to 11% when considering all the patients having received during the study period a medication plan including an orphan prescription, of either an active or a past OMP. Patients treated with OMPs did not differ in terms of age from the globally monitored RD population, as, in both cases, one out of five patient is a pediatric one. This finding differs from the impression derived from the analysis of the EMA OMP designations, according to which children with rare diseases seem to have less benefitted than adults from OMP development (Aartsma-Rus et al., 2021). Furthermore, a recent study reported a significant increase in orphan designations for pediatric-onset diseases in the United States (Miller et al., 2021). Our study investigated OMP use across different disease prevalence classes. It has been recently estimated that diseases with a very low prevalence (<9 cases per million) represent 89.1% of all rare diseases, while they explain 2% of the whole population burden attributable to these conditions (Nguengang Wakap et al., 2020). This opposite relationship between the number of rare disease entities and the number of affected patients can be observed when considering OMP use as well.

Another interesting finding of our study is the clustering of OMP use in few rare disease groups. In most of the cases, OMP use occurred in patients affected by the three following groups of diseases: rare neurological diseases, hematologic rare diseases, and inborn errors of metabolism. This distribution reflects epidemiological figures already available for the monitored population (Mazzucato et al., 2018). Nevertheless, it is indicative of the limited access to OMP experienced by selected groups of RD patients. A recent study estimated survival in a population of RD patients similar to the one considered in the present analysis (Gorini et al., 2021). Patients suffering from rare neurologic diseases, respiratory diseases, and diseases of the skin and subcutaneous tissue had a lower survival compared to the general population. Considering the inborn errors of metabolism group, 15% and 20% of patients with lysosomal storage disorders did not survive at 5 and 10 years from diagnosis, respectively. These findings, combined with data derived from our study, can help understand which therapeutic areas are most in need of research investment, including, but not limited to, OMP development. Real-world data, besides the impression given by the analysis of orphan designations, can more precisely identify which groups of diseases, and thus patients, experience a significant lack of therapeutic options and can mostly benefit from investment in pharmacological research. Actually, besides some impressive successful cases, only a minority of rare diseases patients can access therapies able to significantly modify the natural history of their disease, although highly innovative, as many OMPs are. Furthermore, a scarcity of data referred to the use of other treatments, besides or in combination with OMP, in rare diseases patients exists. Peculiar aspects of complexity deal with the diverse nature of the treatments that potentially can be prescribed to rare diseases patients. In the population under study, medication plans including an OMP present an increasing level of complexity, both in terms of number and nature of prescribed co-treatments. When co-treatments are molecules, a clustering distribution effect in selected ATC classes can be observed. Currently, little is known on the reciprocal interactions between OMP and other prescribed treatments, in terms of both efficacy and safety. This represents an under-investigated area requiring further attention, as our study estimated that in half of the cases OMP use occurred in combination with other treatments, pharmacological and non-pharmacological ones. This occurrence is probably going to become more and more common, due to the emergence of multiple treatment options in specific disease areas. This clustering phenomenon can be observed at the disease level as well. As an example, in the past, in patients diagnosed with phenylketonuria (PKU), the commonest inborn error of metabolism and a disease tested by neonatal screening programs, only one therapeutic approach was available, based on dietary restrictions. The therapeutic landscape for this disease has dramatically changed in recent years and now includes different therapeutic options, to be used in a variety of combinations, according to the patient’s clinical profile. Besides medical foods, these include two approved OMPs, sapropterin and, more recently, pegvaliase (Burlina et al., 2021). Another example is spinal muscular atrophy, the most common genetic cause of death in infancy. In the last decade, new disease-modifying treatments granted with an orphan designation become available and have changed SMA natural history, in particular in SMA1 children. Along with these new therapies, SMA care has conserved its complexity, requiring a high-level expertise for the management of the respiratory and nutritional issues and of other complex healthcare needs presented by these patients (Fauroux et al., 2020; Yerushalmy-Feler et al., 2021). The changes in the therapeutic landscape, including OMPs, require adopting a comprehensive rather than a piecemeal approach when evaluating the relative efficacy and safety profile of different interventions (Yang et al., 2022). This can only be obtained in a real-world setting through data collection systems globally monitoring patients over an adequate time period. Another debated issue when considering the OMP environment is data generation and their ownership (Hollak et al., 2020; Lochmüller et al., 2021). Our study clearly provides reasons to set up and maintain public-funded population-based registries globally monitoring RD patients and supporting their care pathways. The advantages of adopting this approach and of using existing data sources, as RD registries, have been recently outlined, with particular reference to the Italian scenario (Crisafulli et al., 2019). In fact, commonly, OMPs data derive from registries established as a requirement for marketing authorization asked by regulators to the pharmaceutical industries. The limits of this approach are manifold and have been already explored (Hollak et al., 2011; Jonker et al., 2018). First, enrollment of patients into these registries is usually poor. Second, these data collections are usually drug-oriented, while in many rare diseases OMP prescription occurs together with other treatments, pharmaceutical and non-pharmaceutical ones, as outlined by our findings. Third, the majority of post-approval registries required for OMP are set up and funded by industry, with arising issues regarding data access policies and their long-term operation (Hollak et al., 2020; Sirrs et al., 2021). Finally, these data collections are not designed to provide information regarding the use of OMP for indications different from the authorized ones, on which the post-marketing surveillances focus. This is an additional limit, as, according to our findings, off-label use may occur in one out of 10 cases of OMP prescriptions, both in the adult and in the pediatric population.

### Limits

Although this study describes OMP use in a sizable group of rare diseases patients monitored by a long-established population-based registry, it is affected by some limitations that deserve to be mentioned. Some limits are intrinsic to the source of data used. The first one is the possible underestimation due to lack of patients’ enrollment in the registry. This limit is mitigated by the fact that the registry is ongoing since 2002; patient enrollment is linked with exemption from healthcare costs, including high costly therapies, all aspects that guarantee a quite comprehensive coverage. An additional limitation depends on the list of diseases monitored by the registry. The list is not as exhaustive as compared to the Orphanet one; nevertheless, under-representation of some disease groups has been partially overcome in 2017, when a consistent group of RD was added to the national list and thus started to be monitored by the RD registries. Despite this improvement, rare tumors and cystic fibrosis are still not included, as they have been historically monitored by other dedicated registries. As there are OMP available for these therapeutic areas, this limitation warrants particular consideration. Nevertheless, we focused on OMP use in conditions other than oncological ones; also, considering that during the period 2000–2019, the majority of authorized orphan drugs in Europe, 88 out of 129, were for rare non-oncological indications (Hollak et al., 2020). The OMP list has been assembled for the study purposes, and it differs from the one defined by the Italian Medicines Agency, whose inclusion criteria have been revised during the study period. Our list is more comprehensive as it includes past-orphan medicinal products and OMP not fully reimbursed by the National Health System. Thus, the proposed list needs to be updated and adapted to other country-specific situations, for any further use. In the present study, we have analyzed prescriptions registered by clinicians working in centers of expertise entitled to treatment prescription to RD patients. We did not considered data referred to dispensed drugs, truly used by patients. Nevertheless, we think that this limit might affect co-treatment figures rather than OMP ones, for which primary non-compliance is not a relevant issue. In addition, in the study area, completeness and accuracy of treatment recording is high, due to legislation motivating clinicians to record data on prescribed drugs in order to guarantee patients’ access. Another issue to be considered deals with the period under study. In total, two out of the 3 years considered in the present study were interested by the COVID-emergency. This could have affected patients’ care, particularly of RD patients in need of high-specialist care (Chowdhury et al., 2021). Nevertheless, measures to assure continuity of care have been put in place in the study area. Among them are telemedicine use and the automatic renewal of medication plans, in line with the Italian Medicine Agency indications issued during the emergency period, and with actions carried out at the regional level.

## Conclusion

Despite the aforementioned limitations, we consider the provided data on orphan medicinal products use in a defined population a valuable attempt to assume a real-world perspective when tackling OMP-related issues, beyond the evaluation of their economic impact on the healthcare systems. In particular, they highlight the potential of a population-based registry designed to record prescriptions data collected *a priori* and included in medication plans filled by clinicians working in expert RD centers. Available data may be useful in the regulatory pathway, particularly in the price and reimbursement process, to establish the expected number of eligible patients. In fact, these data usually derive from estimations provided by the manufacturer and coming from a multiplicity of different data sources. A recent review evaluating the methodological quality of budget impact analyses for orphan drugs reported that assumptions were mostly made about eligible patients and population size (Abdallah et al., 2021). Real-world data can help overcome these uncertainties, dealing not only with the number of eligible patients but also of patients actually treated with OMP following their marketing authorization, either for approved and for off-label indications.

Data from our registry are routinely used to provide budget impact analyses at the regional level when a new authorized OMP enters the national market. This represents an important example of the added value of a population-based data source, when applied to OMP having an indication for rare diseases, for which an increased prevalence has been reported in defined areas, as is the case of hemoglobinopathies in our region (Colombatti et al., 2008). Registry data are also used to support the designation of centers part of the RD care network, authorized to prescribe selected treatments, when required by the Italian Medicines Agency. As an example, this selection process has been applied for Spinraza^®^ (nusinersen). Furthermore, this example illustrates the role of a population-based registry as a source of data on the entity and the patterns of patients’ switching to other treatments, when more than one OMP becomes available for the same disease or group of diseases, as occurred for SMA patients. Finally, we would like to underline the potential role of RD population–based registry data as a source of external controls, when single arm trials are evaluated by regulatory agencies in treatments approval processes. The potential benefits of this approach have been illustrated, especially when considering subpopulations defined by specific genetic mutations or biomarkers (Burcu et al., 2020).

These preliminary data provide a snapshot of the use of OMP at the population level using an area-based registry monitoring a consistent group of RDs patients, highlighting the value of this data source in generating real-world evidence. Data collected in a real-world setting have the potential to identify which diseases, and thus patients, have less benefit from the advent of OMP so far. Furthermore, in the rapidly evolving RD therapeutic landscape, they can help understand what are the future challenges related to OMP development.

## Data Availability

The datasets generated for this study are not publicly available. Further requests can be directed to: malattierare@regione.veneto.it.
